# Advanced Therapeutic Strategies for Chronic Lung Disease Using Nanoparticle-Based Drug Delivery

**DOI:** 10.3390/jcm5090082

**Published:** 2016-09-20

**Authors:** Ji Young Yhee, Jintaek Im, Richard Seonghun Nho

**Affiliations:** Department of Medicine, University of Minnesota, Minneapolis, MN 55455, USA; jyheephd@umn.edu (J.Y.Y.); imxxx063@umn.edu (J.I.)

**Keywords:** biodistribution, lung diseases, nanoparticle, pulmonary delivery, therapy

## Abstract

Chronic lung diseases include a variety of obstinate and fatal diseases, including asthma, chronic obstructive pulmonary disease (COPD), cystic fibrosis (CF), idiopathic pulmonary fibrosis (IPF), and lung cancers. Pharmacotherapy is important for the treatment of chronic lung diseases, and current progress in nanoparticles offers great potential as an advanced strategy for drug delivery. Based on their biophysical properties, nanoparticles have shown improved pharmacokinetics of therapeutics and controlled drug delivery, gaining great attention. Herein, we will review the nanoparticle-based drug delivery system for the treatment of chronic lung diseases. Various types of nanoparticles will be introduced, and recent innovative efforts to utilize the nanoparticles as novel drug carriers for the effective treatment of chronic lung diseases will also be discussed.

## 1. Introduction

Chronic lung diseases include a wide variety of persistent pulmonary disorders, such as asthma, chronic obstructive pulmonary disease (COPD), cystic fibrosis, pulmonary tuberculosis, idiopathic pulmonary fibrosis (IPF), and lung cancers [1,2]. Some of these diseases are irreversible and often fatal, and no treatments have been shown to be effective for completely restoring lung functions. Approximately 300 million and 210 million people in the world are currently estimated to suffer from the two most prevalent diseases, asthma and COPD, respectively [3,4]. Pulmonary tuberculosis is also a frequently found infectious disease with 8.6 million chronic cases reported in 2012, causing over 1.2 million deaths [1]. IPF is one of the most commonly encountered lethal interstitial lung diseases, and over 2.1 million patients were recorded in previous studies [5,6]. 

Traditional pharmacotherapy for chronic lung diseases can be classified into a few categories according to types of therapeutic agents. A variety of chemical drugs, peptides, antibodies, and genetic molecules (e.g., siRNA, shRNA, and miRNA) have been employed to treat the chronic lung diseases [7,8,9,10]. Unfortunately, most of chronic lung disease cannot be completely cured by pharmacotherapy alone. In cases of asthma, controlling the symptoms is the only available current option. Likewise, steroids, bronchodilators, pirfenidone, and nintedanib are currently used for the management of COPD or IPF, but no effective treatments are available to fully cure these types of diseases. However, pharmacotherapy is still important for chronic lung diseases, and the patients should be properly managed with drugs for their lifetime, or until receiving a lung transplant [11]. 

As an advanced strategy, nanoscale carriers for targeted drug delivery have shown promise in pharmacotherapy. Based on their innate physical properties, nanoparticles can improve the pharmacokinetics of the loaded therapeutics. In addition, current progress in nanoparticles with diverse targeting motifs for the selective delivery to target cells can minimize adverse effects of the drugs. Traditional pharmacotherapy often comes across limitations, and the inappropriate pharmacokinetics and low diffusion of typical drugs frequently result in a low response to the treatment [12,13]. In addition, an efficient vector system is a prerequisite for successful gene therapy, because genetic molecules are not easily delivered into cells without carriers and often degraded in the biological fluids. To address these problems, nanoparticles have been used as drug carriers in recent decades [14,15,16,17]. In this review article, the biophysical properties and pulmonary delivery of nanoparticles will be described first, and current efforts to achieve targeted drug delivery to the lungs will be introduced.

## 2. Concept of Targeted Delivery and in Vivo Behavior of Nanoparticles

Nanoparticles are defined as particles with submicron sizes in diameter. Since the early 2000s, a wide variety of nanoparticles have been developed as drug carriers for biomedical applications. Diverse materials have been used for the fabrication of nanoparticles, and the characteristic properties based on the types of the constituent materials are described in Section 3. Nanoparticles commonly share their unique physical properties due to their submicron sizes, and these characteristics have been exploited for disease-specific drug delivery. Importantly, nanoparticles have a larger surface area than micromaterials (>1 μm) with similar total masses. It allows nanoparticles to have a better chance to have contact with the surrounding tissues and cells, thus increasing the efficiency of cellular delivery [18,19]. Furthermore, systemically injected nanoparticles in vivo generally show enhanced accumulation to the pathological lesions found in tumors, hemorrhagic diseases, and inflammatory diseases [20,21,22]. These findings suggest that nanoparticles have ideal properties to be used as innovative carriers for drug delivery.

### 2.1. Route of Delivery and in Vivo Behavior of Nanoparticles

**Figure 1:**
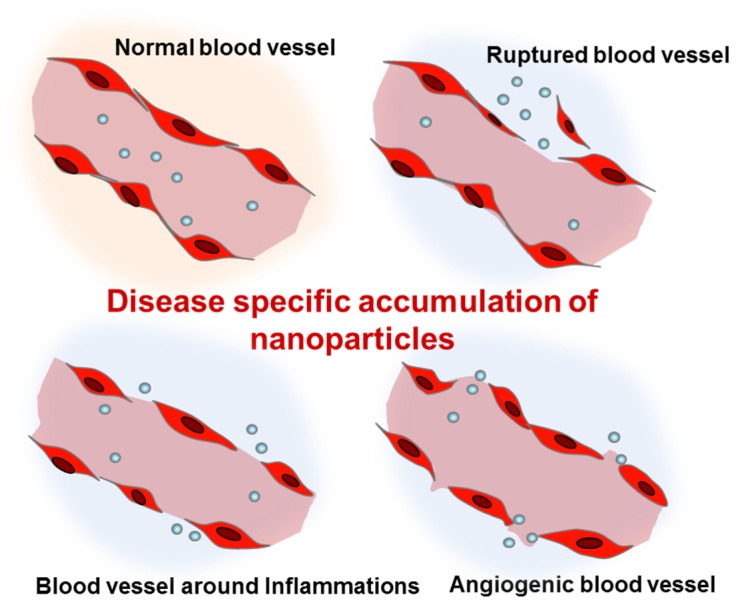
Enhanced permeation of disease-related blood vessels and targeted delivery of intravenously injected nanoparticles.

Nanoparticles can be administered via different routes including intravenous/intraperitoneal injection, oral administration, and pulmonary inhalation. The most typical route of administration has been intravenous injection. Due to their small size, intravenously injected nanoparticles can easily escape from the abnormal blood vessels found in tumors, trauma, hemorrhagic diseases, and chronic inflammation (Figure 1) [23,24,25,26]. In these pathological lesions, the increased local blood flow and the endothelial permeability generally result in the disease-specific accumulation of nanoparticles. In particular, as a result of the impaired lymphatic system of tumor tissues, nanoparticles can show high retention in tumors. Based on the enhanced permeability and retention (EPR) effects [27], cancer has been the most common disease targeted by intravenously injected nanoparticles. Abraxane^®^, the albumin-bound paclitaxel nanoparticle, is the most well-known nanoformulated drug for non-small-cell lung cancer [28]. Abraxane^®^ is an FDA-approved anticancer agent which shows significant retardation of tumor growth. More recently, liposomal nanoformulations for paclitaxel and cisplatin (LEP-ETU and SPI-77) also demonstrated encouraging results for treatment of lung cancers in a phase II clinical trial [29,30].

However, apart from lung cancer, there are still limitations in the use of intravenously injected nanoparticles for the treatment of chronic lung diseases. Noncancerous chronic lung diseases, such as COPD, cystic fibrosis, and IPF may not develop highly permeable blood vessels within the pathological lesions. Since blood vessels with enhanced permeability are an essential prerequisite for the disease-specific accumulation of systemically administered nanoparticles, it might be challenging to achieve an effective nanoparticle delivery for these types of lung disease. Furthermore, proliferative fibrous tissues may cause low diffusion of delivered nanoparticles, preventing them from reaching the target cells. As a result, the in vivo biodistribution of intravenously injected nanoparticles has been rather extensively studied for the purpose of understanding nontargeted delivery to the lung or pulmonary clearance. In a previous study, organ- and tissue-specific biodistribution and the elimination of ultrasmall-sized (<10 nm of hydrodynamic diameter) nanoparticles were investigated in nondiseased mice [31]. The indium arsenide- and zinc sulfide-based ultrasmall-sized nanoparticles were highly distributed in the liver, the kidney, and the intestines within a short time (<l h). However, their accumulations were extremely low in the lung. In other previous studies, larger-sized (100–300 nm) nanoparticles labeled with ^14^C or ^111^In were injected into the mice and rats [32,33,34]. They mainly accumulated in the organs of the reticuloendothelial system, but low accumulation was also found in the lungs [35]. Likewise, some of the nanoparticles are not suitable for lung-targeted delivery, and these types of nanoparticles could not improve the pharmacokinetics of loaded drugs [36,37]. However, prior studies suggest that properly designed nanoparticles which are specialized for the purpose of pulmonary delivery can greatly improve the pharmacokinetics of the drugs. For example, methotrexate-loaded albumin nanoparticle and doxorubicin-loaded solid lipid nanoparticles were highly distributed in the lungs [38,39]. Thus, the biological behaviors of nanoparticles may vary depending on the type and properties of the nanoparticle, and nanoparticles should be carefully designed to improve the pharmacokinetics.

In chronic lung diseases, pulmonary inhalation has been considered as another important route of delivery for nanoparticles [35,40]. Pulmonary inhalation enables higher lung distribution (>3 times higher) of nanoparticles, when compared to the systemic injection or oral administration [41,42]. For inhalation, nanoparticles should be suspended in a gaseous medium. Both dry powder and liquid suspension of nanoparticles are applicable, and specific devices for aerosolization and inhalation are usually required [42,43,44]. A spray drying technique is useful for preparing an inhalable dry powder form of nanoparticles, and carrier matrices such as lactose can be added to the formulation to improve the aerosolization [45,46]. Meanwhile, nanoparticle suspensions can be delivered to the lungs via inhalation using nebulizers [35,47]. Liposomes and solid lipid nanoparticles have been widely used for pulmonary inhalation, because they often preserve their size without aggregation during the aerosolization [42]. Surface modification of the nanoparticles with hydrophilic polymers is also effective in the prevention of the aggregation and opsonization of the nanoparticles in vivo [48,49].

### 2.2. Determinants for the Pulmonary Delivery of Nanoparticles

**Figure 2:**
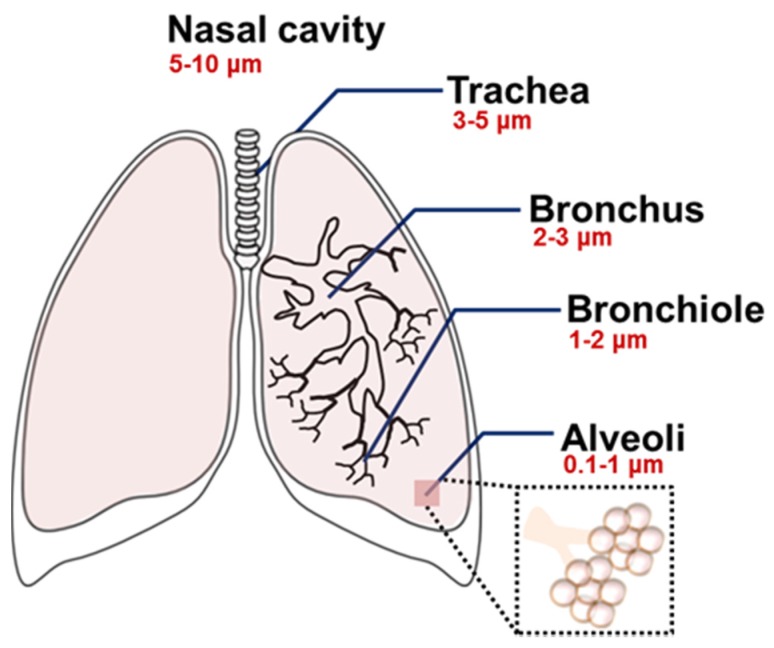
Size-dependent regional deposition of micro- and nanoparticles within the respiratory system after the inhalation. Reproduced from Reference [50] with permission from The Royal Society of Chemistry.

Type of the formulation, composition, shape, and size are all important determinants for the pulmonary delivery of nanoparticles [50,51,52]. In particular, the aerodynamic diameter of nanoparticles is the primary determinant for in vivo distribution of the inhaled nanoparticles [13,42,50,52,53]. The particle size-dependent regional deposition in the lung is illustrated in Figure 2 [50]. In general, the particles larger than 5–6 μm are exhaled, but particles smaller than those sizes can be delivered into the trachea-bronchial region. Ultrafine particles (1–2 μm) are usually deposited in the bronchioles, and particles at the nanoscale (<1 μm) can be delivered to the lower respiratory system including the alveoli. Ultrasmall-sized nanoparticles, such as dendrimers (<20 nm), showed efficient delivery to the alveoli, but they often presented low retention in the lungs due to the rapid penetration into the bloodstream [42,54]. However, the pharmacokinetics of the nanoparticles can be altered after a structural modification. When a dendrimer was modified with various molecular weights of polyethylene glycol (PEG) polymers, the changes of particle size by the PEG modification resulted in a different biodistribution of the dendrimer [55]. Unmodified dendrimers absorbed into the bloodstream with limited lung retention, but modified ones with larger sizes (>78 kDa) accumulated in the lungs [55]. Since there was an evident correlation between the size and absorption/retention of the nanoparticles, the size property should be carefully counted for effective delivery. In addition, it is a necessary step to monitor the actual distribution and the bioavailability of the treated nanoparticles, because nanoparticles often form large aggregates after being released from an aerosol or during the delivery within the respiratory systems [56].

The disease-specific delivery of inhalable nanoparticles may also depend on patient-related or technical factors, such as breathing conditions and aerosolization/inhalation methods [42,50,57,58,59]. In practice, a recent study showed that a vibrating mesh type nebulizer is more effective than a jet nebulizer in terms of pulmonary delivery of a liposomal nanoparticle, because it causes less disruption of the nanoparticle formulations during nebulization [60]. This finding suggests that the delivery efficiency of particles could be even affected by the methods for nanoparticle preparation. In addition, biological barriers and alveolar macrophages should also be considered because these factors can impede the targeted delivery of nanoparticles [35,61].

## 3. Various Nanoparticles for Chronic Lung Diseases

**Figure 3:**
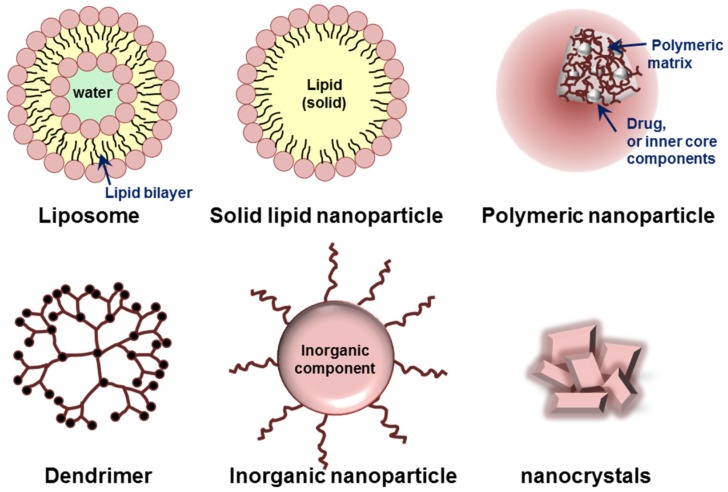
Schematic illustration of various nanoparticles classified based on their building components and characteristic structures.

Various organic or inorganic materials, such as lipids, proteins, synthetic/natural polymers, and metals are currently used for the preparation of nanoparticles [62,63]. Based on their building components and characteristic dimensionality/structures, nanoparticles can be classified into several groups. Liposomes, micelles, polymeric nanoparticles, dendrimers, and inorganic nanoparticles including nanocrystals are typical platforms of nanoparticles (Figure 3). Each type of nanoparticle has its own advantages and limitations for targeted delivery of therapeutics.

### 3.1. Liposomes and Solid Lipid Nanoparticles

Lipid-based materials including cholesterol and phosphatidylcholine are biocompatible, and they have been used for fabrication of nanoparticles from the beginning of nanobiotechnology [62,64]. Liposomes and solid lipid nanoparticles are slightly different in their structure, but they share several benefits as drug carriers. Lipid-based nanoparticles are able to carry large amounts of drugs, and their outer lipid layers contribute to the easy cellular uptake [62,65,66]. For chronic lung diseases, liposomes and solid lipid nanoparticles have a great benefit due to the fact that they are generally stable during aerosolization for inhalation [42]. Based on these several advantages, lipid-based nanoparticles have been studied as a potential carrier system for pulmonary drug delivery over the years. Recently, nanoparticles carrying anticancer drugs, antibiotics, antiasthma agents, and antioxidant agents have been used for the treatment of chronic lung diseases [56,67,68,69,70]. 

### 3.2. Natural and Synthetic Polymer-Based Nanoparticles

Natural and synthetic polymers are both prevalent materials for assembling nanoparticles. Polymers are macromolecules consisting of repeating units of monomers, and the versatile chemical groups of polymers have been utilized for drug conjugation and functionalization of polymer-based nanoparticles. Natural polymers including polysaccharides and proteins are usually extracted from living organisms, and they are highly biocompatible and biodegradable. Synthetic polymers offer multiple benefits in different aspects. They are easily producible and less likely to be biologically contaminated. Each type of polymer has characteristic properties depending on the chemical nature of its building block. For example, PEG polymer has a unique bioinert nature, and it is thus commonly used for the surface modification of nanoparticles [71,72]. PEGylation indeed reduced the opsonization of nanoparticles by the immune cells [62,71], and PEG-coated nanoparticles are also able to penetrate the respiratory mucus due to their muco-inert properties [73]. Meanwhile, as a cationic polymer, polyethyleneimine (PEI) easily binds to nucleotides due to their electrostatic affinity. Based on this property, PEI-based nanoparticles have shown to be effective for gene delivery in chronic lung diseases [62,74,75,76]. As a result of the exclusive features mentioned above, a variety of polymeric nanoparticles has been developed for the treatment of chronic lung diseases including asthma [77,78], tuberculosis [79], and pulmonary hypertension [80].

### 3.3. Dendrimers

Dendrimers are repetitively branched molecules, and they exhibit improved physicochemical properties compared with typical macromolecules. In general, dendrimers are highly monodispersed nanoparticles, and the size and surface functionality of the final formulation are precisely controllable [81]. Dendrimers are capable of carrying a large amount of drugs, and the PEG-modified dendrimer shows favorable pulmonary absorption after inhalation [55,82]. Thus, dendrimers have been widely used for the delivery of therapeutics for chronic lung diseases, and anticancer agents [54], antibiotics [83], and steroids [84] have been reported to be delivered to the lungs by dendrimers.

### 3.4. Inorganic Nanoparticles

Inorganic materials, including gold, iron oxide, and silica, have also been used for building up nanoparticles. Based on the unique plasmonic and magnetic properties, inorganic materials (e.g., gold and iron oxide) generate imaging contrast by computed tomography (CT), magnetic resonance (MR), or positron emission tomography (PET). Consequently, inorganic nanoparticle platforms are also used for diagnostic imaging of diseases [85,86]. Metal nanoparticles, particularly gold nanoparticles, have been extensively studied for gene delivery due to the fact that cationic metal ions easily bind to anionic DNA and RNA molecules [87,88]. In spite of such advantages, inorganic nanoparticles have shown only limited success in treating chronic lung diseases. Although gold nanoparticles were successfully delivered to the alveolar epithelial cells in a COPD mouse model [89], a high degree of toxicity of the nanoparticle still remains a major concern. Besides, positively charged gold nanoparticles possibly bind to negatively charged serum proteins, forming aggregates when they are intravenously injected. A recent study showed that the surface modification of gold nanoparticles with PEG can prevent them from forming aggregates [90]. However, low excretion of gold nanoparticles still hinders their clinical applications and long-term studies. In addition, inorganic nanoparticles are not able to carry large amounts of a chemical drug. Therefore, these limitations should be fully addressed prior to the clinical trials of inorganic nanoparticles [91,92].

## 4. Current Nanomedicine for Chronic Lung Diseases

Nanoparticles were typically administrated via intravenous injection in the early days, and the first generation nanoparticle-based drug delivery was rather focused on the cancer treatment [93]. As a result of the recent advancement of nanotechnologies, the application of nanoparticles has been expanded to other types of lung diseases [8,68,76,79,83,94,95]. Noncancerous lung diseases such as COPD, asthma, and cystic fibrosis can be characterized by severe inflammation and hypersecretion of mucus in the airway. Antibiotics and steroidal/nonsteroidal anti-inflammatory drugs can relieve the symptoms of inflammation, and short-acting β-agonists have been used for the management of COPD [8,9,96]. Nanoparticles are able to enhance the pharmacokinetics of these drugs, potentially providing accurate and controlled drug delivery [97]. In fact, inhalable steroids carrying liposomes and polymeric nanoparticles showed long-lasting drug effects in the fibrotic lungs of animal models [98,99,100]. In addition, curcumin-loaded solid lipid nanoparticles and pirfenidone-loaded poly(lactic-*co*-glycolic) acid (PLGA) nanoparticles were also effective for asthma and pulmonary fibrosis in experimental animal models [95,101]. These studies support the concept that nanoparticles have excellent properties to improve current therapy, and the utilization of nanoparticles is beneficial for the treatment of noncancerous chronic lung diseases by enhancing the bioavailability of drugs.

Recent attempts for gene delivery using nanoparticles are also promising. Cystic fibrosis and α1-antitrypsin deficiency are associated with single gene defects, and most of other chronic lung diseases are also closely related to the failure of the pulmonary defense mechanisms due to genetic disorders [102]. Gene therapy, the correction of dysregulated genes, is expected to provide favorable clinical outcomes in the patients. However, the clinical use of genetic molecules for therapeutic purposes is still limited, as a result of the low efficiency of the delivery to the target cells without vectors. Moreover, RNA molecules are extremely unstable in biological fluid. Therefore, they can be degraded even before reaching the target sites [16,24,75]. As a gene delivery system, nanoparticles indeed enhance the efficiency of cellular delivery and protect genetic molecules from degradation. For example, chitosan nanoparticles carrying interferon (IFN)-γ-plasmid DNA (pDNA) were successfully delivered to mouse lungs to prevent the exacerbation of asthma symptoms [103]. The IFN-γ-pDNA-carrying nanoparticle was injected into the mice before the antigenic challenge, and it significantly prevented allergic CD8+ T cell-related immune responses [103]. Another interesting example is an antimiR-145-carrying lipid nanoparticle [94]. The liposome was delivered to a pulmonary arterial hypertension rat model, and it reduced the density of occlusive vascular lesions and repaired heart structure [94]. In spite of these encouraging results, single gene targeted therapy often fails as a result of the incompleteness in breaking down the whole disease-related pathways [104]. The onset and the progression of diseases are usually not caused by a single molecular abnormality. Therefore, current efforts focus on the preparation of nanoparticles carrying multiple-genetic materials to address this limitation. 

More recently, a new class of drugs has brought attention as an innovative therapeutic strategy [8]. Mepolizumab, an interleukin-5 (IL-5) antibody, substantially reduced asthma exacerbations in human patients with eosinophilic asthma [105,106]. CXC chemokine receptor (CXCR)-2 antagonist also showed promising results in preventing inflammation by inhibiting the inflammatory mediator IL-8 [107]. In addition, phosphoinositide 3-kinase (PI3K) inhibitors, which are involved in the differentiation of alveolar epithelial stem cells, were employed to repair pulmonary alveoli in COPD animal models [108]. Perhaps, the new class of drugs in combination with patient-friendly nanoparticles will likely contribute to more efficient treatment or management of asthma and COPD. 

Lastly, nanoparticles can also provide molecular imaging of chronic lung diseases. A previous study demonstrated that antibody-conjugated superparamagnetic iron oxide (SPIO) nanoparticles were used for noninvasive MR imaging of macrophage subpopulations [109]. In lipopolysaccharide (LPS)-induced COPD mouse models, anti-CD86 and anti-CD206 antibody-conjugated SPIO nanoparticles were intrapulmonary instilled, and each type of SPIO nanoparticles showed specific affinity to the M1 and M2 subpopulation of macrophages. Surface modification with targeting moiety offers selective delivery of nanoparticles to the target cells, and targeted delivery of nanoparticles contributes to high contrast molecular imaging, as well as to targeted therapy.

## 5. Perspectives

Over the last decade, various types of nanoparticles have been developed. Recent technical advances in medicine and nanobiotechnology provide a strong possibility that targeted therapy using nanoparticles is promising for the treatment of chronic lung diseases. However, there are a few issues still remaining for their successful clinical application. Most of all, potential organ toxicity is a major concern in pulmonary medicine. Prior studies have shown that inhaled nanoparticles could be a risk factor for pulmonary inflammation and fibrosis [18,110]. In particular, inorganic nanoparticles should be carefully used because they are not biodegradable and not easily excreted from the site of delivery. In addition, potential lung injury caused by the interaction of nanoparticles and the immune systems of the patients should also be considered. Although it is hard to predict the in vivo toxicity of nanoparticles, carefully selected and designed nanoparticles may minimize such limitations. 

Another foremost obstacle would be the mucus barrier in the respiratory tract. Hypersecretion of mucus in the airway is a common symptom of chronic lung disease, and it is considered a major physical barrier for the delivery of inhaled nanoparticles. Highly viscous sputum in cystic fibrosis is adhesive to the nanoparticles and subsequently impedes their penetration into the underlying respiratory epithelium. To overcome this, various strategies have been attempted to enhance the penetration of nanoparticles to the lung tissues. Shielding the surface of nanoparticles by PEG modification, encapsulating mucolytic agents, and using mannitol to create a local gradient of osmotic pressure improved their penetration to the mucus and showed enhanced delivery to the underlying targeted lung tissues [111,112,113,114,115]. A recent review article described the mucus-related barriers and the overcoming strategies for the targeted delivery of inhaled nanoparticles [116], and it is recommended for further information. 

In this article, we discussed nanoparticle-based drug delivery for chronic lung diseases to provide a better understanding of current advanced therapeutic strategies. Although clinical applications of nanoparticles in the field of respiratory medicine are still in the early stage, innovative nanomaterials are currently being developed. Despite the remaining hurdles, scientific progress in medical science and technical improvement in nanobiotechnology will find highly reliable nanoparticles, which will lead to achieve the complete cure of chronic lung diseases.

## References

[1] World Health Organization (2013). Global Tuberculosis Report 2013.

[2] World Health Organization (2007). Global Surveillance, Prevention and Control of Chronic Respiratory Diseases: A Comprehensive Approach.

[3] Halbert R.J., Natoli J.L., Gano A., Badamgarav E., Buist A.S., Mannino D.M. (2006). Global burden of COPD: Systematic review and meta-analysis. Eur. Respir. J..

[4] Masoli M., Fabian D., Holt S., Beasley R., Global Initiative for Asthma P (2004). The global burden of asthma: Executive summary of the GINA Dissemination Committee report. Allergy.

[5] Ley B., Collard H.R. (2013). Epidemiology of idiopathic pulmonary fibrosis. Clin. Epidemiol..

[6] Raghu G., Weycker D., Edelsberg J., Bradford W.Z., Oster G. (2006). Incidence and prevalence of idiopathic pulmonary fibrosis. Am. J. Respir. Crit. Care Med..

[7] Ruppert C., Schmidt R., Grimminger F., Suzuki Y., Seeger W., Lehr C.M., Gunther A. (2002). Chemical coupling of a monoclonal antisurfactant protein-B antibody to human urokinase for targeting surfactant-incorporating alveolar fibrin. Bioconjug. Chem..

[8] Durham A.L., Caramori G., Chung K.F., Adcock I.M. (2016). Targeted anti-inflammatory therapeutics in asthma and chronic obstructive lung disease. Transl. Res..

[9] Baker K.E., Bonvini S.J., Donovan C., Foong R.E., Han B., Jha A., Shaifta Y., Smit M., Johnson J.R., Moir L.M. (2014). Novel drug targets for asthma and COPD: Lessons learned from in vitro and in vivo models. Pulm. Pharmacol. Ther..

[10] Fujita Y., Takeshita F., Kuwano K., Ochiya T. (2013). RNAi Therapeutic Platforms for Lung Diseases. Pharmaceuticals.

[11] Meyer K.C. (2014). Diagnosis and management of interstitial lung disease. Transl. Respir. Med..

[12] Burhan E., Ruesen C., Ruslami R., Ginanjar A., Mangunnegoro H., Ascobat P., Donders R., van Crevel R., Aarnoutse R. (2013). Isoniazid, rifampin, and pyrazinamide plasma concentrations in relation to treatment response in Indonesian pulmonary tuberculosis patients. Antimicrob. Agents Chemother..

[13] Carvalho T.C., Peters J.I., Williams R.O. (2011). Influence of particle size on regional lung deposition—What evidence is there?. Int. J. Pharm..

[14] Bahadori M., Mohammadi F. (2012). Nanomedicine for respiratory diseases. Tanaffos.

[15] Smola M., Vandamme T., Sokolowski A. (2008). Nanocarriers as pulmonary drug delivery systems to treat and to diagnose respiratory and non respiratory diseases. Int. J. Nanomed..

[16] Di Gioia S., Trapani A., Castellani S., Carbone A., Belgiovine G., Craparo E.F., Puglisi G., Cavallaro G., Trapani G., Conese M. (2015). Nanocomplexes for gene therapy of respiratory diseases: Targeting and overcoming the mucus barrier. Pulm. Pharmacol. Ther..

[17] Ratemi E., Sultana Shaik A., Al Faraj A., Halwani R. (2016). Alternative approaches for the treatment of airway diseases: Focus on nanoparticle medicine. Clin. Exp. Allergy.

[18] Buzea C., Pacheco I.I., Robbie K. (2007). Nanomaterials and nanoparticles: Sources and toxicity. Biointerphases.

[19] Oberdorster G., Oberdorster E., Oberdorster J. (2005). Nanotoxicology: An emerging discipline evolving from studies of ultrafine particles. Environ. Health Perspect..

[20] Thurman J.M., Serkova N.J. (2015). Non-invasive imaging to monitor lupus nephritis and neuropsychiatric systemic lupus erythematosus. F1000Research.

[21] Janib S.M., Moses A.S., MacKay J.A. (2010). Imaging and drug delivery using theranostic nanoparticles. Adv. Drug Deliv. Rev..

[22] Yhee J.Y., Son S., Kim S.H., Park K., Choi K., Kwon I.C. (2014). Self-assembled glycol chitosan nanoparticles for disease-specific theranostics. J. Control Release.

[23] Chung C.Y., Yang J.T., Kuo Y.C. (2013). Polybutylcyanoacrylate nanoparticles for delivering hormone response element-conjugated neurotrophin-3 to the brain of intracerebral hemorrhagic rats. Biomaterials.

[24] Lee S.J., Lee A., Hwang S.R., Park J.S., Jang J., Huh M.S., Jo D.G., Yoon S.Y., Byun Y., Kim S.H., Kwon I.C., Youn I., Kim K. (2014). TNF-alpha gene silencing using polymerized siRNA/thiolated glycol chitosan nanoparticles for rheumatoid arthritis. Mol. Ther..

[25] Sharma H.S., Ali S.F., Dong W., Tian Z.R., Patnaik R., Patnaik S., Sharma A., Boman A., Lek P., Seifert E., Lundstedt T. (2007). Drug delivery to the spinal cord tagged with nanowire enhances neuroprotective efficacy and functional recovery following trauma to the rat spinal cord. Ann. N. Y. Acad. Sci..

[26] Matsumura Y., Maeda H. (1986). A new concept for macromolecular therapeutics in cancer chemotherapy: Mechanism of tumoritropic accumulation of proteins and the antitumor agent smancs. Cancer Res..

[27] Ngoune R., Peters A., von Elverfeldt D., Winkler K., Putz G. (2016). Accumulating nanoparticles by EPR: A route of no return. J. Control Release.

[28] Gupta N., Hatoum H., Dy G.K. (2014). First line treatment of advanced non-small-cell lung cancer—Specific focus on albumin bound paclitaxel. Int. J. Nanomed..

[29] Chang H.I., Yeh M.K. (2012). Clinical development of liposome-based drugs: Formulation, characterization, and therapeutic efficacy. Int. J. Nanomed..

[30] Ait-Oudhia S., Mager D.E., Straubinger R.M. (2014). Application of pharmacokinetic and pharmacodynamic analysis to the development of liposomal formulations for oncology. Pharmaceutics.

[31] Choi H.S., Ipe B.I., Misra P., Lee J.H., Bawendi M.G., Frangioni J.V. (2009). Tissue- and organ-selective biodistribution of NIR fluorescent quantum dots. Nano Lett..

[32] Gipps E.M., Arshady R., Kreuter J., Groscurth P., Speiser P.P. (1986). Distribution of polyhexyl cyanoacrylate nanoparticles in nude mice bearing human osteosarcoma. J. Pharm. Sci..

[33] Rolland A., Collet B., Le Verge R., Toujas L. (1989). Blood clearance and organ distribution of intravenously administered polymethacrylic nanoparticles in mice. J. Pharm. Sci..

[34] Bazile D.V., Ropert C., Huve P., Verrecchia T., Marlard M., Frydman A., Veillard M., Spenlehauer G. (1992). Body distribution of fully biodegradable [14C]-poly(lactic acid) nanoparticles coated with albumin after parenteral administration to rats. Biomaterials.

[35] Azarmi S., Roa W.H., Lobenberg R. (2008). Targeted delivery of nanoparticles for the treatment of lung diseases. Adv. Drug Deliv. Rev..

[36] Chen J.H., Wang L., Ling R., Li Y., Wang Z., Yao Q., Ma Z. (2004). Body distribution of nanoparticle-containing adriamycin injected into the hepatic artery of hepatoma-bearing rats. Dig. Dis. Sci..

[37] Yeh T.K., Lu Z., Wientjes M.G., Au J.L. (2005). Formulating paclitaxel in nanoparticles alters its disposition. Pharm. Res..

[38] Zara G.P., Cavalli R., Fundaro A., Bargoni A., Caputo O., Gasco M.R. (1999). Pharmacokinetics of doxorubicin incorporated in solid lipid nanospheres (SLN). Pharmacol. Res..

[39] Santhi K., Dhanaraj S.A., Koshy M., Ponnusankar S., Suresh B. (2000). Study of biodistribution of methotrexate-loaded bovine serum albumin nanospheres in mice. Drug Dev. Ind. Pharm..

[40] Lee W.H., Loo C.Y., Traini D., Young P.M. (2015). Inhalation of nanoparticle-based drug for lung cancer treatment: Advantages and challenges. Asian J. Pharm. Sci..

[41] Savla R., Minko T. (2013). Nanotechnology approaches for inhalation treatment of fibrosis. J. Drug Target..

[42] Kuzmov A., Minko T. (2015). Nanotechnology approaches for inhalation treatment of lung diseases. J. Control Release.

[43] Lehofer B., Bloder F., Jain P.P., Marsh L.M., Leitinger G., Olschewski H., Leber R., Olschewski A., Prassl R. (2014). Impact of atomization technique on the stability and transport efficiency of nebulized liposomes harboring different surface characteristics. Eur. J. Pharm. Biopharm..

[44] Jiang H.L., Hong S.H., Kim Y.K., Islam M.A., Kim H.J., Choi Y.J., Nah J.W., Lee K.H., Han K.W., Chae C. (2011). Aerosol delivery of spermine-based poly(amino ester)/Akt1 shRNA complexes for lung cancer gene therapy. Int. J. Pharm..

[45] Kawashima Y., Serigano T., Hino T., Yamamoto H., Takeuchi H. (1998). A new powder design method to improve inhalation efficiency of pranlukast hydrate dry powder aerosols by surface modification with hydroxypropylmethylcellulose phthalate nanospheres. Pharm. Res..

[46] Sham J.O., Zhang Y., Finlay W.H., Roa W.H., Lobenberg R. (2004). Formulation and characterization of spray-dried powders containing nanoparticles for aerosol delivery to the lung. Int. J. Pharm..

[47] Rudokas M., Najlah M., Alhnan M.A., Elhissi A. (2016). Liposome Delivery Systems for Inhalation: A Critical Review Highlighting Formulation Issues and Anticancer Applications. Med. Princ. Pract..

[48] Winterhalter M., Frederik P.M., Vallner J.J., Lasic D.D. (1997). Stealth(R) liposomes: From theory to product. Adv. Drug Deliv. Rev..

[49] Allen T.M. (1998). Liposomal drug formulations. Rationale for development and what we can expect for the future. Drugs.

[50] Kumar A., Chen F., Mozhi A., Zhang X., Zhao Y., Xue X., Hao Y., Zhang X., Wang P.C., Liang X.J. (2013). Innovative pharmaceutical development based on unique properties of nanoscale delivery formulation. Nanoscale.

[51] Pilcer G., Amighi K. (2010). Formulation strategy and use of excipients in pulmonary drug delivery. Int. J. Pharm..

[52] Van Rijt S.H., Bein T., Meiners S. (2014). Medical nanoparticles for next generation drug delivery to the lungs. Eur. Respir. J..

[53] Muralidharan P., Malapit M., Mallory E., Hayes D., Mansour H.M. (2015). Inhalable nanoparticulate powders for respiratory delivery. Nanomedicine.

[54] Kaminskas L.M., McLeod V.M., Ryan G.M., Kelly B.D., Haynes J.M., Williamson M., Thienthong N., Owen D.J., Porter C.J. (2014). Pulmonary administration of a doxorubicin-conjugated dendrimer enhances drug exposure to lung metastases and improves cancer therapy. J. Control Release.

[55] Ryan G.M., Kaminskas L.M., Kelly B.D., Owen D.J., McIntosh M.P., Porter C.J. (2013). Pulmonary administration of PEGylated polylysine dendrimers: Absorption from the lung versus retention within the lung is highly size-dependent. Mol. Pharm..

[56] Paranjpe M., Muller-Goymann C.C. (2014). Nanoparticle-mediated pulmonary drug delivery: A review. Int. J. Mol. Sci..

[57] Chan J.G., Wong J., Zhou Q.T., Leung S.S., Chan H.K. (2014). Advances in device and formulation technologies for pulmonary drug delivery. AAPS PharmSciTech.

[58] Faiyazuddin M., Mujahid M., Hussain T., Siddiqui H.H., Bhatnagar A., Khar R.K., Ahmad F.J. (2013). Aerodynamics and deposition effects of inhaled submicron drug aerosol in airway diseases. Recent Pat. Inflamm. Allergy Drug Discov..

[59] d’Angelo I., Perfetto B., Costabile G., Ambrosini V., Caputo P., Miro A., d’Emmanuele di Villa Bianca R., Sorrentino R., Donnarumma G., Quaglia F., Ungaro F. (2016). Large Porous Particles for Sustained Release of a Decoy Oligonucelotide and Poly(ethylenimine): Potential for Combined Therapy of Chronic Pseudomonas aeruginosa Lung Infections. Biomacromolecules.

[60] Elhissi A.M.A., Faizi M., Naji W.F., Gill H.S., Taylor K.M.G. (2007). Physical stability and aerosol properties of liposomes delivered using an air-jet nebulizer and a novel micropump device with large mesh apertures. Int. J. Pharm..

[61] Niven R.W. (1995). Delivery of biotherapeutics by inhalation aerosol. Crit. Rev. Ther. Drug Carr. Syst..

[62] Yhee J.Y., Son S., Kim N., Choi K., Kwon I.C. (2014). Theranostic applications of organic nanoparticles for cancer treatment. Mrs Bull..

[63] Kim T., Hyeon T. (2014). Applications of inorganic nanoparticles as therapeutic agents. Nanotechnology.

[64] Estella-Hermoso de Mendoza A., Campanero M.A., Mollinedo F., Blanco-Prieto M.J. (2009). Lipid nanomedicines for anticancer drug therapy. J. Biomed. Nanotechnol..

[65] Nassimi M., Schleh C., Lauenstein H.D., Hussein R., Hoymann H.G., Koch W., Pohlmann G., Krug N., Sewald K., Rittinghausen S., Braun A., Muller-Goymann C. (2010). A toxicological evaluation of inhaled solid lipid nanoparticles used as a potential drug delivery system for the lung. Eur. J. Pharm. Biopharm..

[66] Nassimi M., Schleh C., Lauenstein H.D., Hussein R., Lubbers K., Pohlmann G., Switalla S., Sewald K., Muller M., Krug N., Muller-Goymann C.C., Braun A. (2009). Low cytotoxicity of solid lipid nanoparticles in in vitro and ex vivo lung models. Inhal. Toxicol..

[67] Patel A.R., Chougule M.B., Ian T., Patlolla R., Wang G., Singh M. (2013). Efficacy of aerosolized celecoxib encapsulated nanostructured lipid carrier in non-small cell lung cancer in combination with docetaxel. Pharm. Res..

[68] Elhissi A.M.A., Islam M.A., Arafat B., Taylor M., Ahmed W. (2010). Development and characterisation of freeze-dried liposomes containing two anti-asthma drugs. Micro Nano Lett..

[69] Hoesel L.M., Flierl M.A., Niederbichler A.D., Rittirsch D., McClintock S.D., Reuben J.S., Pianko M.J., Stone W., Yang H., Smith M. (2008). Ability of antioxidant liposomes to prevent acute and progressive pulmonary injury. Antioxid. Redox Signal..

[70] Liu C., Shi J., Dai Q., Yin X., Zhang X., Zheng A. (2015). In vitro and in vivo evaluation of ciprofloxacin liposomes for pulmonary administration. Drug Dev. Ind. Pharm..

[71] Jokerst J.V., Lobovkina T., Zare R.N., Gambhir S.S. (2011). Nanoparticle PEGylation for imaging and therapy. Nanomedicine.

[72] Van Vlerken L.E., Vyas T.K., Amiji M.M. (2007). Poly(ethylene glycol)-modified nanocarriers for tumor-targeted and intracellular delivery. Pharm. Res..

[73] Schuster B.S., Suk J.S., Woodworth G.F., Hanes J. (2013). Nanoparticle diffusion in respiratory mucus from humans without lung disease. Biomaterials.

[74] Bivas-Benita M., Romeijn S., Junginger H.E., Borchard G. (2004). PLGA-PEI nanoparticles for gene delivery to pulmonary epithelium. Eur. J. Pharm. Biopharm..

[75] Patnaik S., Gupta K.C. (2013). Novel polyethylenimine-derived nanoparticles for in vivo gene delivery. Expert Opin. Drug Deliv..

[76] Ungaro F., d’Angelo I., Coletta C., d’Emmanuele di Villa Bianca R., Sorrentino R., Perfetto B., Tufano M.A., Miro A., La Rotonda M.I., Quaglia F. (2012). Dry powders based on PLGA nanoparticles for pulmonary delivery of antibiotics: Modulation of encapsulation efficiency, release rate and lung deposition pattern by hydrophilic polymers. J. Control Release.

[77] Seong J.H., Lee K.M., Kim S.T., Jin S.E., Kim C.K. (2006). Polyethylenimine-based antisense oligodeoxynucleotides of IL-4 suppress the production of IL-4 in a murine model of airway inflammation. J. Gene Med..

[78] Surti N., Naik S., Bagchi T., Dwarkanath B.S., Misra A. (2008). Intracellular delivery of nanoparticles of an antiasthmatic drug. AAPS PharmSciTech.

[79] Pandey R., Sharma A., Zahoor A., Sharma S., Khuller G.K., Prasad B. (2003). Poly (dl-lactide-co-glycolide) nanoparticle-based inhalable sustained drug delivery system for experimental tuberculosis. J. Antimicrob. Chemother..

[80] Kimura S., Egashira K., Chen L., Nakano K., Iwata E., Miyagawa M., Tsujimoto H., Hara K., Morishita R., Sueishi K., Tominaga R., Sunagawa K. (2009). Nanoparticle-mediated delivery of nuclear factor kappaB decoy into lungs ameliorates monocrotaline-induced pulmonary arterial hypertension. Hypertension.

[81] Kesharwani P., Jain K., Jain N.K. (2014). Dendrimer as nanocarrier for drug delivery. Prog. Polym. Sci..

[82] Bharatwaj B., Mohammad A.K., Dimovski R., Cassio F.L., Bazito R.C., Conti D., Fu Q., Reineke J., da Rocha S.R. (2015). Dendrimer nanocarriers for transport modulation across models of the pulmonary epithelium. Mol. Pharm..

[83] Bellini R.G., Guimaraes A.P., Pacheco M.A., Dias D.M., Furtado V.R., de Alencastro R.B., Horta B.A. (2015). Association of the anti-tuberculosis drug rifampicin with a PAMAM dendrimer. J. Mol. Graph. Model..

[84] Inapagolla R., Guru B.R., Kurtoglu Y.E., Gao X., Lieh-Lai M., Bassett D.J., Kannan R.M. (2010). In vivo efficacy of dendrimer-methylprednisolone conjugate formulation for the treatment of lung inflammation. Int. J. Pharm..

[85] Cho E.C., Glaus C., Chen J., Welch M.J., Xia Y. (2010). Inorganic nanoparticle-based contrast agents for molecular imaging. Trends Mol. Med..

[86] Swierczewska M., Lee S., Chen X. (2011). Inorganic nanoparticles for multimodal molecular imaging. Mol. Imaging.

[87] Ding Y., Jiang Z., Saha K., Kim C.S., Kim S.T., Landis R.F., Rotello V.M. (2014). Gold nanoparticles for nucleic acid delivery. Mol. Ther..

[88] Capek I., Tiwari A. (2015). DNA Engineered Noble Metal Nanoparticles: Fundamentals and State-of-the-Art of Nanobiotechnology.

[89] Geiser M., Quaile O., Wenk A., Wigge C., Eigeldinger-Berthou S., Hirn S., Schaffler M., Schleh C., Moller W., Mall M.A., Kreyling W.G. (2013). Cellular uptake and localization of inhaled gold nanoparticles in lungs of mice with chronic obstructive pulmonary disease. Part. Fibre Toxicol..

[90] Alkilany A.M., Murphy C.J. (2010). Toxicity and cellular uptake of gold nanoparticles: What we have learned so far?. J. Nanopart. Res..

[91] Chen H.W., Su S.F., Chien C.T., Lin W.H., Yu S.L., Chou C.C., Chen J.J., Yang P.C. (2006). Titanium dioxide nanoparticles induce emphysema-like lung injury in mice. FASEB J..

[92] Shi H., Magaye R., Castranova V., Zhao J. (2013). Titanium dioxide nanoparticles: A review of current toxicological data. Part Fibre Toxicol..

[93] Chandolu V., Dass C.R. (2013). Treatment of lung cancer using nanoparticle drug delivery systems. Curr. Drug Discov. Technol..

[94] McLendon J.M., Joshi S.R., Sparks J., Matar M., Fewell J.G., Abe K., Oka M., McMurtry I.F., Gerthoffer W.T. (2015). Lipid nanoparticle delivery of a microRNA-145 inhibitor improves experimental pulmonary hypertension. J. Control Release.

[95] Wang W., Zhu R., Xie Q., Li A., Xiao Y., Li K., Liu H., Cui D., Chen Y., Wang S. (2012). Enhanced bioavailability and efficiency of curcumin for the treatment of asthma by its formulation in solid lipid nanoparticles. Int. J. Nanomed..

[96] Montuschi P., Malerba M., Santini G., Miravitlles M. (2014). Pharmacological treatment of chronic obstructive pulmonary disease: From evidence-based medicine to phenotyping. Drug Discov. Today.

[97] Da Silva A.L., Santos R.S., Xisto D.G., Alonso Sdel V., Morales M.M., Rocco P.R. (2013). Nanoparticle-based therapy for respiratory diseases. Anais da Academia Brasileira de Ciencias.

[98] Joshi M., Misra A.N. (2001). Pulmonary disposition of budesonide from liposomal dry powder inhaler. Methods Find Exp. Clin. Pharmacol..

[99] Konduri K.S., Nandedkar S., Duzgunes N., Suzara V., Artwohl J., Bunte R., Gangadharam P.R. (2003). Efficacy of liposomal budesonide in experimental asthma. J. Allergy Clin. Immunol..

[100] Matsuo Y., Ishihara T., Ishizaki J., Miyamoto K., Higaki M., Yamashita N. (2009). Effect of betamethasone phosphate loaded polymeric nanoparticles on a murine asthma model. Cell Immunol..

[101] Trivedi R., Redente E.F., Thakur A., Riches D.W., Kompella U.B. (2012). Local delivery of biodegradable pirfenidone nanoparticles ameliorates bleomycin-induced pulmonary fibrosis in mice. Nanotechnology.

[102] Kolb M., Martin G., Medina M., Ask K., Gauldie J. (2006). Gene therapy for pulmonary diseases. Chest.

[103] Kong X., Hellermann G.R., Zhang W., Jena P., Kumar M., Behera A., Behera S., Lockey R., Mohapatra S.S. (2008). Chitosan Interferon-gamma Nanogene Therapy for Lung Disease: Modulation of T-Cell and Dendritic Cell Immune Responses. Allergy Asthma Clin. Immunol..

[104] Hopkins A.L. (2008). Network pharmacology: The next paradigm in drug discovery. Nat. Chem. Biol..

[105] Nair P., Pizzichini M.M., Kjarsgaard M., Inman M.D., Efthimiadis A., Pizzichini E., Hargreave F.E., O’Byrne P.M. (2009). Mepolizumab for prednisone-dependent asthma with sputum eosinophilia. N. Engl. J. Med..

[106] Pavord I.D., Korn S., Howarth P., Bleecker E.R., Buhl R., Keene O.N., Ortega H., Chanez P. (2012). Mepolizumab for severe eosinophilic asthma (DREAM): A multicentre, double-blind, placebo-controlled trial. Lancet.

[107] Boppana N.B., Devarajan A., Gopal K., Barathan M., Bakar S.A., Shankar E.M., Ebrahim A.S., Farooq S.M. (2014). Blockade of CXCR2 signalling: A potential therapeutic target for preventing neutrophil-mediated inflammatory diseases. Exp. Biol. Med..

[108] Horiguchi M., Oiso Y., Sakai H., Motomura T., Yamashita C. (2015). Pulmonary administration of phosphoinositide 3-kinase inhibitor is a curative treatment for chronic obstructive pulmonary disease by alveolar regeneration. J. Control Release.

[109] Al Faraj A., Shaik A.S., Afzal S., Al Sayed B., Halwani R. (2014). MR imaging and targeting of a specific alveolar macrophage subpopulation in LPS-induced COPD animal model using antibody-conjugated magnetic nanoparticles. Int. J. Nanomed..

[110] Bonner J.C. (2010). Nanoparticles as a potential cause of pleural and interstitial lung disease. Proc. Am. Thorac. Soc..

[111] Ibrahim B.M., Park S., Han B., Yeo Y. (2011). A strategy to deliver genes to cystic fibrosis lungs: A battle with environment. J. Control Release.

[112] Suk J.S., Lai S.K., Wang Y.Y., Ensign L.M., Zeitlin P.L., Boyle M.P., Hanes J. (2009). The penetration of fresh undiluted sputum expectorated by cystic fibrosis patients by non-adhesive polymer nanoparticles. Biomaterials.

[113] Lai S.K., O’Hanlon D.E., Harrold S., Man S.T., Wang Y.Y., Cone R., Hanes J. (2007). Rapid transport of large polymeric nanoparticles in fresh undiluted human mucus. Proc. Natl. Acad. Sci. USA.

[114] Broughton-Head V.J., Smith J.R., Shur J., Shute J.K. (2007). Actin limits enhancement of nanoparticle diffusion through cystic fibrosis sputum by mucolytics. Pulm. Pharmacol. Ther..

[115] Yang Y., Tsifansky M.D., Shin S., Lin Q., Yeo Y. (2010). Mannitol-Guided Delivery of Ciprofloxacin in Artificial Cystic Fibrosis Mucus Model. Biotechnol. Bioeng..

[116] Kim N., Duncan G.A., Hanes J., Suk J.S. (2016). Barriers to inhaled gene therapy of obstructive lung diseases: A review. J. Control Release.

